# Retainment of Poly(3-hydroxybutyrate-*co*-3-hydroxyhexanoate) Properties from Oil-Fermented *Cupriavidus necator* Using Additional Ethanol-Based Defatting Process

**DOI:** 10.3390/polym17152058

**Published:** 2025-07-28

**Authors:** Tae-Rim Choi, Gaeun Lim, Yebin Han, Jong-Min Jeon, Shashi Kant Bhatia, Hyun June Park, Jeong Chan Joo, Hee Taek Kim, Jeong-Jun Yoon, Yung-Hun Yang

**Affiliations:** 1 Advanced Materials Program, Department of Biological Engineering, College of Engineering, Konkuk University, Hwayang-dong, Gwangjin-gu, Seoul 05029, Republic of Korea; srim1004@gmail.com (T.-R.C.); lge0919@naver.com (G.L.); hanyebin3@gmail.com (Y.H.); shashikonkukuni@konkuk.ac.kr (S.K.B.); 2 Green Circulation R&D Department, Korea Institute of Industrial Technology (KITECH), Cheonan 31056, Republic of Korea; j2pco@kitech.re.kr (J.-M.J.); jjyoon@kitech.re.kr (J.-J.Y.); 3 Institute for Ubiquitous Information Technology and Applications, Konkuk University, Seoul 05029, Republic of Korea; 4 Department of Biotechnology, Duksung Women’s University, Seoul 10370, Republic of Korea; junehpark@duksung.ac.kr; 5 Department of Chemical Engineering, Kyung Hee University, Yongin-si 17104, Republic of Korea; jcjoo@khu.ac.kr; 6 Department of Food Science and Technology, Chungnam National University, Daejeon 34134, Republic of Korea; heetaek@cnu.ac.kr

**Keywords:** poly(3-hydroxybutyrate-*co*-3-hydroxyhexanoate), recovery, *Cupriavidus necator*

## Abstract

Engineering of *Cupriavidus necator* could enable the production of various polyhydroxyalkanoates (PHAs); particularly, poly(3-hydroxybutyrate-*co*-3-hydroxyhexanoate) (P(3HB-*co*-3HH)), a biopolymer with enhanced mechanical and thermal properties compared to poly(3-hydroxybutyrate) (PHB), can be efficiently produced from vegetable oils. However, challenges remain in the recovery process, particularly in removing residual oil and minimizing degradation of the polymer structure during extraction steps. This study investigated the effects of ethanol-based defatting on the recovery and polymeric properties of P(3HB-*co*-3HH). The proposed method involves the addition of ethanol to the cell broth to effectively remove residual oil. Ethanol improved the separation of microbial cells from the broth, thereby streamlining the downstream recovery process. Using ethanol in the washing step increased the recovery yield and purity to 95.7% and 83.4%, respectively (compared to 87.4% and 76.2% for distilled water washing), representing improvements of 8.3% and 7.2%. Ethanol washing also resulted in a 19% higher molecular weight compared to water washing, indicating reduced polymer degradation. In terms of physical properties, the elongation at break showed a significant difference: 241.9 ± 27.0% with ethanol washing compared to water (177.7 ± 10.3%), indicating ethanol washing retains flexibility. Overall, an ethanol washing step for defatting could simplify the recovery steps, increase yield and purity, and retain mechanical properties, especially for P(3HB-*co*-3HH) from oils.

## 1. Introduction

Polyhydroxyalkanoate (PHA), a bacterial storage polyester, is currently gaining considerable attention owing to its biodegradability and potential to replace petroleum-derived polymers, such as polypropylene [1,2,3]. *Cupriavidus necator* is commonly employed for the production of microbial PHAs as it efficiently accumulates a substantial amount of poly(3-hydroxybutyrate) (PHB) and is easy to cultivate. Researchers have been investigating the production of different copolymers from *C. necator* to improve their properties and potential applications [4,5]. A well-studied PHA is poly(3-hydroxybutyrate-*co*-3-hydroxyhexanoate) (P(3HB-*co*-3HH)); it exhibits a lower melting temperature, lower Young’s modulus, and increased elongation at break than PHB [6,7], suggesting that it is a tougher and more flexible plastic [8]. *C. necator* contains both an enoyl-CoA hydratase (*phaJ*) and PHA synthase (*phaC*) and can produce P(3HB-*co*-3HH) from various carbon sources. Generally, PHA production can be achieved using a variety of carbon sources, including pure substrates (glucose, fructose, and glycerol) and complex compounds (lignocellulosic biomass) [9,10]. For copolymer P(3HB-*co*-3HH) production, vegetable oil is considered the best feedstock, as its metabolism through the β-oxidation pathway provides precursors for the 3HH monomer unit [11,12,13].

Several methods have been developed for the downstream recovery of PHAs, including solvent extraction [14,15,16], chemical or enzymatic digestion [17,18,19], and simultaneous digestion and extraction [20,21]. PHA recovery from cultures necessitates pretreatment steps, such as cell harvesting, cell disruption, and washing; however, P(3HB-*co*-3HH) production from oils requires additional steps to remove residual oils [22]. This is partially achievable using hexane. Furthermore, prior to the drying step, pretreatment of cells by stirring or flushing with mildly polar solvents, such as methanol, ethanol, or acetone, is advantageous [23]. This process removes lipids from the cells, resulting in a pure, odorless, and colorless product. Methanol and ethanol are effective in the pretreatment of cells, enhancing both the recovery and purity of PHA [24]. This treatment step compromises the cell envelope, rendering the subsequent PHA solvent extraction more efficient [25,26].

Notably, recent studies have proposed advanced downstream methods for PHA recovery—such as supercritical carbon dioxide extraction and ionic-liquid-based processes—to improve efficiency and selectivity in polymer recovery [27,28,29]. However, these approaches often require specialized equipment and costlier reagents [30]. In contrast, ethanol is widely available, relatively safe (with lower toxicity and flammability than many organic solvents), and cost-effective, making it a practical choice for industrial PHA processing [31,32]. When explicitly comparing ethanol defatting to conventional solvents like hexane or to emerging methods, ethanol has advantages in terms of cost, safety, and sustainability [23,33,34]

Repeated dissolution and precipitation of the polymer can considerably increase the purity of the final product [35]. Consequently, the pretreatment process could be an alternative for enhancing product purity and minimizing recovery steps. Although these solvents have been used to remove residual oil and disrupt cell membranes, their effects on the thermal and physical properties of PHA have not been fully studied, given that pretreatment steps result in excessive waste generation, rendering the methods environmentally unfriendly and uneconomical [23,36]. Moreover, the use of hard surfactants, strong bases, or sodium hypochlorite leads to polymer decomposition [20]; thus, this process warrants careful consideration, and the impact of the defatting step should be thoroughly evaluated.

In this study, alterations in physical and thermal properties resulting from additional treatment with various solvents, including hexane and short-chain alcohols such as ethanol, were examined for the first time. Furthermore, based on the elucidated polymer properties, a downstream process for P(3HB-*co*-3HH) was proposed.

## 2. Materials and Methods

### 2.1. Microorganism and Production of Biomass Containing P(3HB-co-3HH)

*Cupriavidus necator* H16 was used for cultivation. The pCB81 vector (harboring *phaC* from *Rhodococcus aetherovorans* I24, *phaJ* from *Pseudomonas aeruginosa* PAO1, and *phaA* from *C. necator* H16) [37] was introduced into cells via conjugation with *Escherichia coli* S17-1 harboring pCB81 [38].

*C. necator* H16 containing pCB81 was cultivated using a dissolved oxygen-stat fed-batch culture in a 5 L jar fermenter (CNS, Daejeon, Korea). The minimal media contained 4.0 g/L NaH_2_PO_4_, 4.6 g/L Na_2_HPO_4_, 0.45 g/L K_2_SO_4_, 0.39 g/L MgSO_4_, 62 mg/L CaCl_2_, and 1 mL/L of a trace element solution with an initial pH of 6.8 [39]. The trace element solution contained 15 g/L FeSO_4_⋅7H_2_O, 2.4 g/L MnSO_4_⋅H_2_O, 2.4 g/L ZnSO_4_⋅7H_2_O, and 0.48 g/L CuSO_4_⋅5H_2_O dissolved in 0.1 M hydrochloric acid. Bean oil and (NH_4_)_2_SO_4_ were used as the carbon and nitrogen sources, respectively, with fed-batch cultivation implemented by the addition of 200 mL of bean oil 18 h post-inoculation [40]. At the end of the fermentation period, the culture broth was stored for further experiments. Before storage, 1 mL of the culture broth was harvested; washed twice with distilled water (DW) and hexane; and analyzed to set the control parameters for dry cell weight, PHA content, and 3HH mole fraction. If not otherwise specified in the text, all reagents were purchased from Sigma-Aldrich (St. Louis, MO, USA).

### 2.2. P(3HB-co-3HH) Biomass Pretreatment

Biomass was recovered by centrifuging 25 mL of culture broth in 50 mL Falcon tubes at 3500 rpm for 15 min. From the culture broth sample, the dry cell weight, PHA content, and 3HH mole fraction were determined by lyophilization at −80 °C for 18 h and subsequent analysis by gas chromatography (GC), as previously described [41].

Various P(3HB-*co*-3HH) recovery experiments were performed. Initially, 25 mL of culture broth sample was centrifuged at 3500 rpm for 20 min to collect biomass. For the pretreatment process, 50 mL of different wash solvents, such as DW, hexane, ethanol, and methanol, were added. After suspension of the biomass pellet, it was stored at 25 °C for 1 h. Biomass was recovered by centrifugation at 3500 rpm for 20 min, and the supernatant was collected to determine the wash rate. The collected wash solvent was evaporated, and the weight of the residual oil was measured. Wash rate was calculated using the residual lipids present in the unwashed supernatant as a control. Hexane (50 mL) was then added to the centrifuged sample. Following hexane washing, the biomass was recovered by centrifugation and re-washed with water. The final biomass pellet obtained was subjected to lyophilization at −80 °C for 18 h or oven-drying at 80 °C for up to 48 h. Biomass pretreatment was conducted in duplicate for all data presented with error bars. The pretreated biomass was analyzed, and the measured data for the culture broth, recovery rate, and purity of the copolymers were calculated [21].

### 2.3. PHA Extraction, Film Formation, and Scanning Electron Microscopy (SEM) Analysis

Lyophilized biomass (1 g) was dissolved in 50 mL of chloroform for 1 h at 55 °C. The solution was filtered through Whatman filter paper to remove cell debris and other impurities. The filtered chloroform solution was poured into a round glass Petri dish. At room temperature (approximately 25 °C), in a fume-hood system, the chloroform was allowed to completely evaporate (up to 18 h) until a white- to brown-colored PHA film formed. The PHA film was used for further analyses [40].

Backscattered electron images were acquired using a TM4000Plus SEM instrument (Hitachi, Tokyo, Japan) at 5 kV [42,43].

### 2.4. PHA Quantification

PHA was quantified and characterized using GC–mass spectrometry (GC-MS), as previously described [44]. The culture broth (1 mL) was centrifuged, and the resulting pellet was washed twice with 1 mL of DW and 1 mL of hexane. The washed cells were then transferred into a glass vial for overnight lyophilization, and the dry cell weight was measured. Next, equal volumes of chloroform and 15% (*v*/*v*) H_2_SO_4_/85% methanol solution (2 mL total volume) were added to a glass vial, and methanolysis was performed at 100 °C for 2 h, followed by cooling to room temperature. A 1 mL aliquot of deionized water was added to the methyl ester solution, and the mixture was vortexed twice for 10 s each. The chloroform layer was then transferred to a microtube containing anhydrous Na_2_SO_4_ to remove any residual water. Filtered 1 μL aliquots were injected into the GC-MS (PerkinElmer, Waltham, MA, USA) equipped with a triple-axis detector and an Elite 5 ms column (30 mm length, 0.25 mm internal diameter, and 0.25 µm film). Helium was used as the carrier gas at a flow rate of 48.3 mL/min. The injector temperature was set at 280 °C. The temperature programs for the column and oven were set as follows: an increase of 10 °C for 1 min to 120 °C at 15 °C/min, followed by a 15 min hold, and then an increase to 300 °C at 10 °C/min, which was then held for another 15 min. The NIST/EPA/NIH library was used to identify the methylated PHAs [4].

### 2.5. Gel Permeation Chromatography Analysis

Gel permeation chromatography (GPC) was used to determine the number-average molecular weight (Mn), weight-average molecular weight (Mw), and polydispersity index (PDI) of the polymer. GPC was performed using an HPLC system (YoungIn Chromass, Seoul, Korea) comprising a loop injector (Rheodyne 7725i; Rheodyne, Rohnert Park, CA, USA), a dual-headed isocratic pump system (YL9112; YoungIn Chromass, Seoul, Korea), a column oven (YL9131; YoungIn Chromass, Seoul, Korea) with three columns (K-G 4A, guard column; K-804 8.0 × I.D. × 300 mm; and K-805, 8.0 × 300 mm; Shodex), and a refractive index detector (YL9170). Chloroform was used as the mobile phase at a flow rate of 1 mL/min at 40 °C. The injection volume was 20 µL. Polystyrene standards (Shodex, Tokyo, Japan) ranging from 5000 to 2,000,000 Da were used to calculate the molecular weight and construct the calibration curve [4].

### 2.6. Universal Testing Machine Analysis

A universal testing machine (UTM), EZ-LX (Shimadzu, Kyoto, Japan), was used to measure the tensile strength, Young’s modulus, and elongation at the break of each sample [20]. The samples were cut into 10 mm × 60 mm pieces, and the gauge length was defined accordingly. All tests were conducted in at least triplicate for each sample at a crosshead speed of 10 mm/min. Elongation at break (EL) was calculated using Equation (1):

*EL* = (*d_after_* − *d_before_*)/*d_before_* × 100 (1)

where *d* represents the distance between the grips holding the sample before and after the sample breaks.

### 2.7. Differential Scanning Calorimetry Analysis

A PHA sample (5 mg) was loaded onto a Hitachi NEXTA DSC200 (Hitachi, Tokyo, Japan) instrument in a sample pan. Samples were analyzed using the following temperature protocol: initiation at 30 °C; the temperature was increased at a rate of 50 °C/min to 190 °C, and held for 10 min. Next, the temperature was reduced from 190 °C to −60 °C at 50 °C/min and held for 10 min. This was followed by a second heating cycle, which was increased from −60 °C to 190 °C at 10 °C/min, held for 10 min, and then decreased from 190 °C to −60 °C at 10 °C/min.

## 3. Results and Discussion

### 3.1. Design of PHA Recovery Method for Oil-Fermented Biomass

The *C. necator* strain harboring the pCB81 vector was cultured in a 5 L jar fermenter with a working volume of 2 L for 165 h with bean oil as the carbon source. The final biomass had a dry cell weight of 177.2 g/L, 87.17% PHA content, and a 14.19 mol% of a 3-HH mole fraction as accumulated PHA. After fermentation, the biomass was stored at −80 °C until further recovery experiments were conducted.

**Figure 1:**
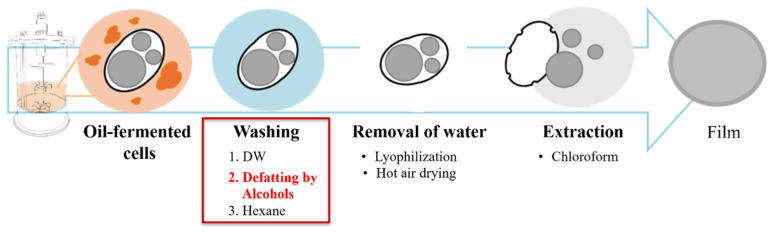
Downstream processes of PHA production. PHA recovery requires several steps. Following oil fermentation, the culture broth contains residual oils and media components. Separation using centrifugation and washing steps with distilled water, alcohols, and hexane is required to remove these oils and components. To remove the remaining water, drying steps such as lyophilization or hot air drying are common. Chloroform is used to dissolve and concentrate PHA. These methods and complex downstream processing result in PHA powder or film. Red text indicates key suggestion points in the downstream process of oil-fermented biomass, specifically related to replacing the hexane wash.

To determine the impact of washing solvent application on the recovery method and to compare its efficacy with the conventional PHA recovery method (Figure 1), a series of preliminary experiments was conducted, including lipid washing as a liquid–liquid extraction, normal washing with DW, short-chain alcohols (ethanol and methanol), and hexane. This pretreatment not only removed the residual lipids in the cells but also compromised the cell membrane, thereby facilitating subsequent PHA recovery steps [24].

**Figure 2:**
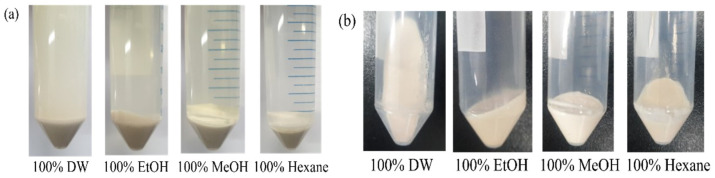
PHA biomass pretreatment procedures. The biomass properties change according to the wash solvent used. (**a**) Biomass pellets washed with distilled water, ethanol, methanol, and hexane exhibited different pellet patterns when centrifuged. (**b**) When the supernatant was decanted, the water- and hexane-washed pellets easily flowed despite the same centrifugation conditions.

When various solvents (DW, ethanol, methanol, and hexane) were used for washing and centrifugation, the shapes of the pellet and supernatant differed. The DW sample contained an opaque supernatant and a brown-yellow pellet. The ethanol and methanol samples contained clear supernatant and solid-packed pellets. Hexane produced a clear supernatant, but the pellet was loose (Figure 2a,b). The ethanol and methanol samples formed solid pellets, whereas the DW and hexane sample pellets flowed out easily as they were loosely packed (Figure 2b). The enhanced pellet integrity observed with ethanol and methanol treatments can be attributed to their ability to disrupt exopolymeric substances (EPSs), which reduce the viscosity of the culture broth, thereby improving cell aggregation and sedimentation during centrifugation [23].

**Figure 3:**
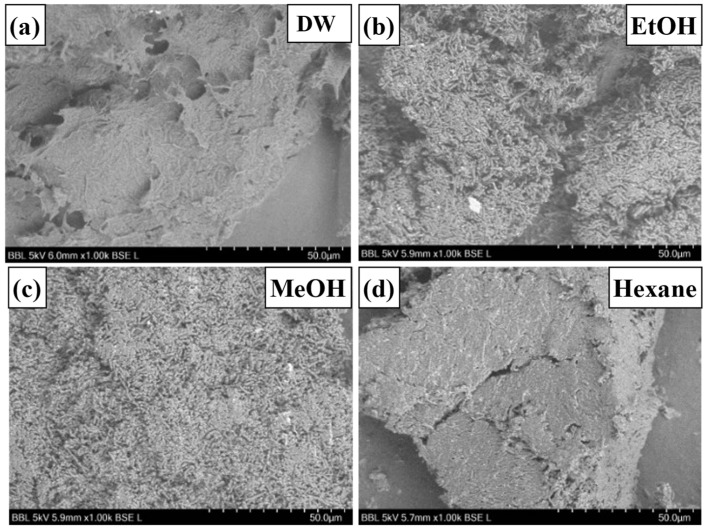
SEM image of the surface of washed and lyophilized fermented cells. The surface images of pretreated and lyophilized samples differed based on the solvents used for washing. SEM image of the surface of (**a**) distilled water wash, (**b**) ethanol wash, (**c**) methanol wash, and (**d**) hexane wash.

The lyophilized powders of all the samples showed a similar texture. SEM analysis of water- and hexane-pretreated samples (Figure 3a,d) showed a tightly packed floccule-like structure, which represented the presence of a large amount of residual oil, whereas in samples pretreated with ethanol and methanol (Figure 3b,c), microbial cells were easily observed. Hexane, while effective in lipid removal, did not significantly affect the EPS or cell surface characteristics, resulting in looser pellet formation [28,45,46]

The wash rate for each wash step was calculated based on the number of lipids in the no-wash sample (used as a control). The supernatant was collected from all the samples and analyzed for residual lipids. Supernatants of the no-wash control contained 5.89 g/L of residual lipids. DW samples contained 3.96 g/L of residual lipids, resulting in a 33.08% wash rate, whereas ethanol- and hexane-pretreated sample supernatants exhibited 70.46% and 51.95% wash rates for the first wash, respectively (Table 1). The ethanol-washed sample was the most efficient at 71.11%. This result suggests that short-chain alcohols have a positive effect on lipid removal from the cell biomass. Therefore, biomasses with various wash solvents were also analyzed to determine the property changes.

### 3.2. Changes in Physical and Thermal Properties Resulting from Wash Processes and Lyophilization

**Figure 4:**
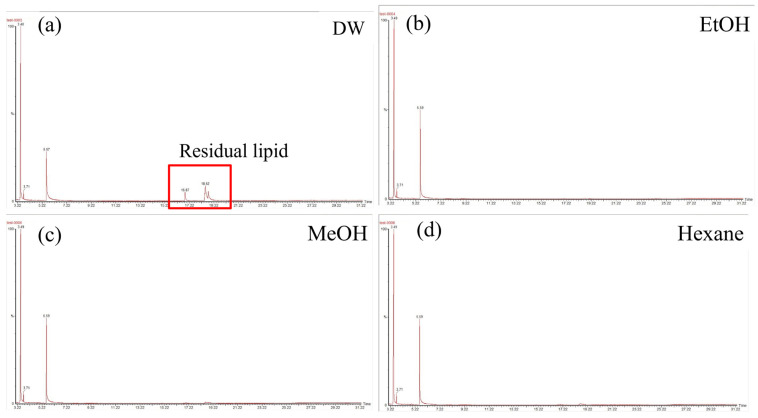
GC chromatogram results. To determine the purity and recovery rate of each pretreated sample, GC analysis was conducted. GC chromatogram image of (**a**) distilled water wash, (**b**) ethanol wash, (**c**) methanol wash, and (**d**) hexane wash. The major peaks are 3-hydroxybutyrate and 3-hydroxyhexanoate for the quantification of PHA, and other peaks represent residual lipids.

GC enabled the analysis of 3-HB and 3-HH peaks for quantification and residual lipids as impurities. The DW samples had clear and large lipid peaks, including palmitoleic and oleic acids, which are the major components of bean oil (Figure 4a). The other three samples using different wash solvents exhibited very small lipid peaks (Figure 4b–d). The purity and recovery rate were also calculated from the biomass, derivatized for quantification, and the PHA calibration curve was constructed using authentic PHB and P(3HB-co-3HH) samples. The washing solvent did not affect the molar fraction of 3-HH in P(3HB-*co*-3HH) (Table 2). The recovery rate of the washed cells was calculated by dividing the amount of PHA by the amount of PHA in the control sample. The ethanol wash exhibited higher values of 95% recovery and 83% purity than the hexane wash, which demonstrated a 91% recovery rate and 79% purity.

The mechanical analyses presented in Table 3 demonstrate that solvent selection during washing significantly influences the tensile strength, elongation at break, and Young’s modulus of P(3HB-*co*-3HH). Notably, the ethanol-washed samples exhibited substantially higher elongation values (~241%) compared to water-washed (~98%) or hexane-washed (~44%) counterparts while maintaining similar tensile strength (6.5–7.1 MPa) and modulus (200–227 MPa). Mechanically, elongation at break is sensitive to microstructural integrity: higher values indicate fewer chain scissions and more ductile behavior. The improved ductility in ethanol-washed samples suggests that ethanol not only removes residual lipids effectively but also minimizes moisture-induced hydrolysis during drying—retaining a long polymer chain length and preventing embrittlement [47,48]. By contrast, water-washed and hexane-washed polymers likely retain residual water or undergo incomplete lipid removal, leading to chain cleavage and formation of microvoids during lyophilization—reflected in lower elongation and a similar tensile modulus [33,49]. Furthermore, short-chain alcohols like ethanol are known to better disrupt cell membranes and remove non-PHA materials before drying, resulting in more homogeneous polymer films and enhanced mechanical resilience [49].

Thus, the Table 3 data support the conclusion that ethanol washing preserves mechanical integrity by preventing both hydrolytic degradation and structural defects, leading to significantly improved elongation while maintaining strength and stiffness. This highlights the dual role of ethanol in both defatting and moisture control during PHA recovery.

### 3.3. Sequential Washing and Drying Effects on PHA Recovery

As in previous studies, serial washing steps, including DW, hexane, and ethanol, and their effects on the polymer properties were observed [37,39]. Basic information on the recovery rate, 3HH mole fraction, and purity is presented in Table 4. The DW + EtOH + hexane + lyophilization sequence showed the highest recovery yield (95.1%) and purity (94.0%), indicating that the inclusion of ethanol in the washing step significantly enhanced polymer recovery and purity. In contrast, the DW + DW + hexane + lyophilization method resulted in the lowest recovery yield (61.6%) and purity (78.4%), suggesting that washing with DW alone is insufficient for effective oil removal.

The physical properties of the fully processed PHA varied significantly depending on the solvent used for the washing steps before lyophilization. An ethanol wash resulted in the highest tensile strength (6.9 ± 0.3 MPa) and elongation at break (241.9 ± 27.0%), indicating the significantly enhanced flexibility and durability of the polymer compared to water and hexane washes and lyophilization (Table 5). Conversely, hexane washing followed by lyophilization showed the lowest elongation at break (140.7 ± 16.5%), suggesting reduced flexibility. Young’s modulus, which reflects the stiffness of the material, was highest for ethanol plus lyophilization (180.5 ± 10.7 MPa), whereas hexane plus lyophilization resulted in the lowest stiffness (147.5 ± 11.2 MPa). These differences highlight the critical role of solvent selection in tailoring the mechanical properties of PHA for specific applications.

The molecular weights and PDI values of recovered P(3HB-*co*-3HH) polymers after various washing steps are summarized in Table 6. The ethanol-washed sample exhibited the highest number-average molecular weight (Mn = 85.8 kDa) and the lowest PDI (2.29), suggesting a more uniform molecular weight distribution and less degradation. In contrast, the hexane-washed sample showed the lowest Mn (69.0 kDa) and relatively high PDI (2.53), indicating more severe polymer chain scission and a broader molecular weight distribution. Compared to reports on P(3HB-co-3HH) molecular weights ranging from 300 to 500 kDa for industrial-grade materials [37,48,50], all recovered samples in this study showed lower values. This discrepancy is primarily due to a prolonged fermentation time (>120 h), which can lead to intracellular degradation processes such as depolymerase activity [37]. The consistent difference across all washing treatments confirms that polymer chain length is determined during fermentation and that washing and recovery mainly preserve rather than substantially alter polymer molecular weight. Importantly, ethanol washing demonstrated a clear advantage in preserving higher Mn and narrower PDI over both water and hexane treatments. This effect is hypothesized to stem from ethanol’s superior capacity to remove intracellular moisture [51,52], reducing hydrolytic cleavage of ester bonds during the drying and extraction steps [26,53,54,55]. Water- and hexane-washed samples retained more moisture, as supported by the observed lower elongation at break and higher PDI, suggesting partial hydrolysis and chain scission. Additionally, although the weight-average molecular weight (Mw) values of ethanol- and water-washed samples were similar (196.6 and 204.9 kDa, respectively), the ethanol-treated sample showed a lower PDI, indicating a more uniform molecular architecture. This reinforces the interpretation that ethanol pretreatment offers both polymer integrity protection and a homogenizing effect on polymer chain distribution.

In summary, ethanol washing not only improved polymer recovery yield and purity but also contributed to maintaining a polymer molecular weight closer to its biosynthetic maximum under the given fermentation conditions, emphasizing its dual functional role in both defatting and dehydration during downstream processing.

The changes in the thermal properties of P(3HB-*co*-3HH) recovered using different washing solvents are listed in Table 7. All samples had a melting temperature ranging from 170.8 to 172.7 °C. Although the 3-HHx mole fraction was measured as approximately 14%, the melting temperatures of the samples were much higher than those of other reports. When the 3-HHx mole fraction increased from 3% to 11%, the melting temperature decreased from 149.6 to 126 °C [48,50]. However, the crystallization temperature (Tc) was similar to that of other reports at approximately 50–60 °C. The literature reports the Tm for P(3HB-*co*-3HH) typically around 120–150 °C, depending on 3HH content [47,48]. In this study, delta H was notably low, indicating low crystallinity. We hypothesize that fractionated polymer structures with long PHB-rich blocks and short 3HH segments, which could explain the high Tm with low crystallinity [56]. Further studies, including ^13^C NMR analysis and fractionation, are required to confirm this hypothesis. However, this study focuses on evaluating changes in thermal properties resulting from downstream processing methods, rather than characterizing the intrinsic thermal behavior of the starting polymer itself. Therefore, this study discusses the main conclusions drawn regarding the impact of washing solvents and drying methods on PHA recovery and properties.

The proposed mechanism—that ethanol’s dehydration effect prevents hydrolysis of the PHA—is supported by the observed maintenance of molecular weight and mechanical properties in ethanol-treated samples. Water residues in the biomass are known to hinder extraction and lead to hydrolytic reduction in the PHA molar mass. Ethanol’s ability to remove water likely reduces this hydrolysis. Studies on PHA degradation indicate that hydrolytic cleavage of ester bonds is accelerated in the presence of water and acidic catalysts [50,57,58]. By contrast, dehydrated conditions limit such reactions. Indeed, previous work has shown that PHA chain scission is significantly reduced under low-humidity and -solvent conditions. References [50,53,54] discuss the importance of controlling moisture during drying to preserve PHA integrity. Some reports explicitly examine the hydrolysis kinetics of P(3HB-*co*-3HH) [50,53], underscoring our hypothesis. Therefore, ethanol likely acts as an “antisolvent” for moisture—a concept similarly applied in other sustainable PHA processes—thereby minimizing degradation.

### 3.4. Downstream Processing: Drying Methods and Cost Implications

Regardless of the extraction solvent used, microbial PHA-rich biomass must generally undergo a drying step before polymer extraction [33]. This can be achieved by either lyophilization (freeze-drying) or thermal treatment. Water residues in the biomass hinder the efficiency of the extraction process, leading to hydrolytic reduction in the PHA molar mass [57,58]. Compared to lyophilization, thermal treatment can significantly shorten the polyester chains, and controlling the complex thermal hydrolysis of P(HB-*co*-3HH) is challenging [53,58]. Lyophilization, being a more “sound” technique, mitigates these chain scission reactions [59]. However, considering the operational costs of commercial PHA production, thermal drying is less capital-intensive than lyophilization, which requires more complex equipment [60]. Notably, freeze-drying has been recognized as extremely expensive and is typically only used when necessary for high-value products [61]. Thus, from an economic standpoint, a process that allows for safe thermal drying could significantly reduce costs.

Our results showed that oven drying (thermal drying) water- or hexane-washed biomass caused a critical deterioration in mechanical properties (Table 8). For instance, tensile strengths in oven-dried, water-washed PHA fell to ~1.6 MPa (vs. ~6.8 MPa for lyophilized), and elongation dropped from ~98% to 27%. In contrast, ethanol-washed biomass subjected to oven drying retained much better properties (elongation, ~98%; tensile, ~6.4 MPa). This suggests that ethanol pretreatment allows for the use of cheaper thermal drying without severe polymer degradation by protecting PHA chains. With ethanol washing, moisture is removed sufficiently such that thermal drying does not induce as much hydrolysis. The cost savings are twofold: first, improved yield/purity from ethanol wash (95% yield, 83% purity) means less material loss; second, replacing lyophilization with oven drying can cut both capital and energy costs. A recent process analysis notes that the capital investment for freeze-drying systems is substantially higher than for thermal dryers [61], and the per-batch energy consumption requires 30% to 10 times more energy than oven drying for equivalent volumes [57,62,63,64,65]. Additionally, more than 90% of the ethanol used in the washing process can be recovered through distillation or rotary evaporation and reused in subsequent downstream processing [66,67]. Therefore, defatting with ethanol and drying with ovens is promising for reducing costs and enhancing the overall sustainability of the process by minimizing solvent waste generation for the PHA downstream process.

The physical properties of the oven-dried PHA showed reduced mechanical performance compared to those of the lyophilized samples.

Ethanol-dried PHA exhibited the highest tensile strength (3.8 ± 0.3 MPa) and Young’s modulus (100.5 ± 6.9 MPa), reflecting its superior durability and stiffness (Table 9). However, its elongation at break (78.7 ± 7.5%) was lower than that of the water-dried samples (71.9 ± 11.1%), suggesting a slight trade-off in flexibility. The hexane-dried samples demonstrated lower tensile strength and comparable stiffness (101.5 ± 10.8 MPa) but had the lowest elongation at break, indicating diminished elasticity and mechanical robustness.

The thermal properties of oven-dried PHA showed slight differences depending on the drying solvent used. Ethanol-dried PHA exhibited the highest melting temperature (172.0 °C) and Tc (94.5 °C), which may reflect enhanced crystalline structure formation during processing (Table 10). Conversely, the hexane-dried samples showed the lowest Tc (54.02 °C), suggesting reduced crystallinity. The glass transition temperatures remained consistent across all samples, similar to lyophilized PHA, indicating that the drying method had minimal impact on this parameter.

The molecular weight analysis of the oven-dried PHA revealed notable variations based on the solvent used. Ethanol drying preserved the highest Mn (65.6 kDa) and Mw (125.4 kDa) values, suggesting minimal polymer degradation (Table 11). In contrast, hexane drying resulted in the lowest Mn (41.0 kDa) and highest PDI (2.883), indicating significant molecular heterogeneity and potential polymer fragmentation. These results highlight the effectiveness of ethanol in maintaining molecular weight integrity during drying compared to water and hexane.

The results showed that the type of wash solvent employed for pretreatment had a considerable effect on property control. Therefore, based on these results, a downstream procedure can be suggested with a wash step using washing solvent or ethanol prior to the dehydration step. Based on the cost comparison of lyophilization and thermal drying, replacing hexane washing—which is commonly used as a downstream step for oil removal—with ethanol washing offers clear cost advantages in terms of solvent price and overall process economics. When we conducted a separate analysis based on the available literature, solvent prices for ethanol were approximately 50% lower than those for hexane (USD 778.82/t for ethanol vs. USD 1568.43/t for hexane) [68]. While hexane may require lower capital expenditure (CAPEX) due to its lower required volume in extraction systems (USD 112.12 million for hexane vs. USD 251.85 million for ethanol), ethanol exhibits a 10.2% lower net present value (NPV) (USD 469.19 million vs. USD 413.53 million) when considering the entire process flow [68].

Taking into account both solvent cost differences and energy savings from drying methods, we estimate that the overall process cost could be reduced by approximately 15–20% when switching from hexane washing combined with lyophilization to ethanol washing combined with oven drying. This is particularly significant considering ethanol’s additional advantages in terms of safety, environmental impact, and regulatory compliance.

## 4. Conclusions

In this study, we systematically evaluated the impact of biomass pretreatment using different wash solvents on the recovery efficiency, purity, and material properties of P(3HB-*co*-3HH) synthesized from oil-based fermentation. Among the tested solvents, ethanol washing demonstrated the most favorable balance between process simplicity, polymer integrity, and economic viability. Specifically, ethanol achieved a recovery yield of approximately 95% and a purity of 83%, outperforming water washing (87% yield; 76% purity) and hexane washing in both quantitative metrics and operational safety. Notably, ethanol-washed P(3HB-*co*-3HH) samples retained superior mechanical properties, including an elongation at break of approximately 242% compared to 178% for water-washed samples while maintaining a comparable molecular weight distribution (Mw ~197 kDa, PDI 2.29). These findings suggested that ethanol not only facilitates lipid removal but also protects polymer chains from hydrolytic degradation by effectively dehydrating the biomass prior to drying. This hypothesis was consistent with the literature on PHA degradation mechanisms, which highlighted the critical role of moisture content in ester bond cleavage during thermal processing. From an industrial perspective, incorporating ethanol washing enabled the replacement of freeze-drying with more cost-effective thermal drying methods, such as oven drying, without compromising polymer quality. Comparative energy and cost analyses indicated that oven drying combined with ethanol pretreatment could reduce operational costs by at least 15–20% relative to conventional lyophilization, offering substantial advantages for scalable PHA production. Furthermore, the ethanol used in washing could be efficiently recovered and recycled via vacuum distillation or rotary evaporation, with solvent recovery efficiencies exceeding 90%, further enhancing the process’s economic and environmental sustainability. Overall, ethanol-based pretreatment represented a scientifically robust and practically viable strategy for large-scale P(3HB-*co*-3HH) recovery. It addressed key industrial bottlenecks related to polymer degradation, process complexity, and production cost. Future research should focus on integrating ethanol recycling systems into continuous production lines and exploring the direct evidence of ethanol washing’s effect on polymers containing biomass to fully optimize process parameters for diverse application requirements.

## Figures and Tables

**Table 1:** Lipid wash rates using different pretreatment solvents.

Sample Wash	Residual Lipids (g/L)	Wash Rate (%)
No wash	5.89 ± 0.04	0 ± 0
DW wash	3.96 ± 0.01	32.77 ± 0.20
EtOH wash	1.74 ± 0.01	70.46 ± 0.02
Hexane wash	2.83 ± 0.00	51.95 ± 0.23
*p*-value	<0.001	<0.001

Statistical significance was determined by one-way ANOVA. *p*-values < 0.05 were considered statistically significant. Error bars represent standard deviation (*n* = 2).

**Table 2:** PHA recovery rate and purity of single-washed biomass.

	Recovery Rate (%)	3HH Mole Fraction (mol%)	Purity (%)
No wash	85.85 ± 0.00	14.98 ± 0.41	74.84 ± 0.00
DW wash	87.40 ± 4.25	14.90 ± 0.17	76.19 ± 3.70
EtOH wash	95.70 ± 3.46	15.03 ± 1.19	83.43 ± 3.01
Hexane wash	91.13 ± 5.89	14.07 ± 0.41	79.44 ± 5.13
*p*-value	0.042	<0.001	0.006

Statistical significance was determined by one-way ANOVA. *p*-values < 0.05 were considered statistically significant. Error bars represent standard deviation (*n* = 2).

**Table 3:** Effect of washing cells with DW, EtOH, hexane, and MeOH for defatting: physical properties of single-wash PHA.

Sample	Tensile Strength (MPa)	Elongation at Break (%)	Young’s Modulus (MPa)
DW wash + lyophilization	6.8 ± 0.5	98.5 ± 7.4	227.2 ± 8.2
EtOH wash + lyophilization	7.1 ± 0.6	241.1 ± 20.9	200.9 ± 8.4
Hexane wash + lyophilization	6.5 ± 0.4	44.2 ± 5.0	221.4 ± 16.5
*p*-value	0.339	<0.001	0.051

Statistical significance was determined by one-way ANOVA. *p*-values < 0.05 were considered statistically significant. Error bars represent standard deviation (*n* = 3).

**Table 4:** Recovery efficiency of different pretreatment solvents and wash steps.

Sample	Recovery Rate (%)	3HH Mole Fraction (mol%)	Purity (%)
DW + DW + hexane + lyophilization	61.6	16.2	78.4
DW + EtOH + hexane + lyophilization	95.1	18.1	94.0
DW + hexane + hexane + lyophilization	77.6	16.5	89.2

**Table 5:** Effect of washing cells with DW, EtOH, and hexane for defatting: physical properties of PHA.

Sample	Tensile Strength (MPa)	Elongation at Break (%)	Young’s Modulus (MPa)
DW + DW + hexane + lyophilization	4.8 ± 0.6	177.7 ± 10.3	159.9 ± 15.8
DW + EtOH + hexane + lyophilization	6.9 ± 0.3	241.9 ± 27.0	180.5 ± 10.7
DW + hexane+ hexane + lyophilization	4.5 ± 0.6	140.7 ± 16.5	147.5 ± 11.2
*p*-value	0.003	0.002	0.061

Statistical significance was determined by one-way ANOVA. *p*-values < 0.05 were considered statistically significant. Error bars represent standard deviation (*n* = 3).

**Table 6:** Effect of washing cells with DW, EtOH, and hexane for defatting: molecular weight of PHA.

Sample	Mn (kDa)	Mw (kDa)	PDI
DW + DW + hexane + lyophilization	78.2	204.9	2.62
DW + EtOH + hexane + lyophilization	85.8	196.6	2.29
DW + hexane + hexane + lyophilization	69.0	174.5	2.53

Mn, number-average molecular weight; Mw, weight-average molecular weight (Mw); PDI, polydispersity index.

**Table 7:** Effect of washing cells with DW, EtOH, and hexane for defatting: thermal properties of PHA.

Sample	T_m_ (°C)	T_c_ (°C)	T_g_ (°C)
DW + DW + hexane + lyophilization	171.7	64.20	−6.93
DW + EtOH + hexane + lyophilization	172.1	58.91	−6.21
DW + hexane + hexane + lyophilization	172.7	60.8	−6.45

T_m_, melting temperature; T_c_, crystallization temperature; T_g_, glass transition temperature.

**Table 8:** Effect of washing cells with DW, EtOH, and hexane with oven drying steps: physical properties of oven-dried PHA.

Sample	Tensile Strength (MPa)	Elongation at Break (%)	Young’s Modulus (MPa)
DW wash + Oven	1.6 ± 0.2	27.0 ± 5.0	62.9 ± 9.5
EtOH wash + Oven	6.4 ± 0.6	98.1 ± 27.6	201.1 ± 14.2
Hexane wash + Oven	2.8 ± 0.1	30.0 ± 4.2	114.7 ± 3.3
*p*-value	<0.001	<0.001	<0.001

Statistical significance was determined by one-way ANOVA. *p*-values < 0.05 were considered statistically significant. Error bars represent standard deviation (*n* = 3).

**Table 9:** Effect of serial washing cells with DW, EtOH, and hexane with oven drying steps: physical properties of oven-dried PHA.

Sample	Tensile Strength (MPa)	Elongation at Break (%)	Young’s Modulus (MPa)
DW + DW + hexane + oven	1.7 ± 0.1	71.9 ± 11.1	62.7 ± 3.0
DW + EtOH + hexane + oven	3.8 ± 0.3	78.7 ± 7.5	100.5 ± 6.9
DW + hexane + hexane + oven	2.4 ± 0.3	59.1 ± 28.1	101.5 ± 10.8
*p*-value	<0.001	0.466	<0.001

Statistical significance was determined by one-way ANOVA. *p*-values < 0.05 were considered statistically significant. Error bars represent standard deviation (*n* = 3).

**Table 10:** Effect of serial washing cells with DW, EtOH, and hexane with oven drying steps: thermal properties of oven-dried PHA.

Sample	T_m_ (°C)	T_c_ (°C)	T_g_ (°C)
DW + DW + hexane + oven	171.3	60.7	−7.41
DW + EtOH + hexane + oven	172.0	94.5	−6.80
DW + hexane + hexane + oven	171.4	54.02	−7.25

T_m_, melting temperature; T_c_, crystallization temperature; T_g_, glass transition temperature.

**Table 11:** Effect of serial washing cells with DW, ETOH, and hexane with oven drying steps: molecular weight of oven-dried PHA.

Sample	Mn (kDa)	Mw (kDa)	PDI
DW + DW + hexane + oven	57.2	99.7	1.74
DW + EtOH + hexane + oven	65.6	125.4	1.91
DW + hexane + hexane + oven	41.0	118.3	2.88

Mn, number-average molecular weight; Mw, weight-average molecular weight (Mw); PDI, polydispersity index.

## Data Availability

All data generated or analyzed during this study are included in this published article.

## Abbreviations

The following abbreviations are used in this manuscript:

DW, distilled water; EPS, exopolymeric substances; GC-MS, GC–mass spectrometry; GPC, gel permeation chromatography; Mn, number-average molecular weight; Mw, weight-average molecular weight; PDI, polydispersity index; PHAs, polyhydroxyalkanoates; P(3HB-*co*-3HH), poly(3-hydroxybutyrate-*co*-3-hydroxyhexanoate); PHB, poly(3-hydroxybutyrate); SEM, scanning electron microscopy; T_m_, melting temperature; T_c_, crystallization temperature; T_g_, glass transition temperature.

## References

[1] Steinbüchel A., Füchtenbusch B. (1998). Bacterial and Other Biological Systems for Polyester Production. Trends Biotechnol..

[2] De Koning G.J.M., Witholt B. (1997). A Process for the Recovery of Poly (Hydroxyalkanoates) from Pseudomonads Part 1: Solubilization. Bioprocess Eng..

[3] Zhang T., Luo X.S., Xu J., Yao X., Fan J., Mao Y., Song Y., Yang J., Pan J., Khattak W.A. (2023). Dry–Wet Cycle Changes the Influence of Microplastics (MPs) on the Antioxidant Activity of Lettuce and the Rhizospheric Bacterial Community. J. Soils Sediments.

[4] Oh S.-J., Kim S., Lee Y., Shin Y., Choi S., Oh J., Bhatia S.K., Joo J.C., Yang Y.-H. (2024). Controlled Production of a Polyhydroxyalkanoate (PHA) Tetramer Containing Different Mole Fraction of 3-Hydroxybutyrate (3HB), 3-Hydroxyvalerate (3 HV), 4 HV and 5 HV Units by Engineered Cupriavidus Necator. Int. J. Biol. Macromol..

[5] Cai F., Lin M., Jin W., Chen C., Liu G. (2023). Biosynthesis of Poly(3-Hydroxybutyrate-Co-3-Hydroxvalerate) from Volatile Fatty Acids by Cupriavidus Necator. J. Basic. Microbiol..

[6] Doi Y., Kitamura S., Abe H. (1995). Microbial Synthesis and Characterization of Poly (3-Hydroxybutyrate-Co-3-Hydroxyhexanoate). Macromolecules.

[7] Noda I., Green P.R., Satkowski M.M., Schechtman L.A. (2005). Preparation and Properties of a Novel Class of Polyhydroxyalkanoate Copolymers. Biomacromolecules.

[8] Bhatia S.K., Gurav R., Choi T.-R., Jung H.-R., Yang S.-Y., Song H.-S., Jeon J.-M., Kim J.-S., Lee Y.-K., Yang Y.-H. (2019). Poly (3-Hydroxybutyrate-Co-3-Hydroxyhexanoate) Production from Engineered Ralstonia Eutropha Using Synthetic and Anaerobically Digested Food Waste Derived Volatile Fatty Acids. Int. J. Biol. Macromol..

[9] Das S., Chandukishore T., Ulaganathan N., Dhodduraj K., Gorantla S.S., Chandna T., Gupta L.K., Sahoo A., Atheena P.V., Raval R. (2024). Sustainable Biorefinery Approach by Utilizing Xylose Fraction of Lignocellulosic Biomass. Int. J. Biol. Macromol..

[10] Jo S.Y., Lim S.H., Lee J.Y., Son J., Choi J.I., Park S.J. (2024). Microbial Production of Poly(3-Hydroxybutyrate-Co-3-Hydroxyvalerate), from Lab to the Shelf: A Review. Int. J. Biol. Macromol..

[11] Crisafi F., Valentino F., Micolucci F., Denaro R. (2022). From Organic Wastes and Hydrocarbons Pollutants to Polyhydroxyalkanoates: Bioconversion by Terrestrial and Marine Bacteria. Sustainability.

[12] Brigham C.J., Budde C.F., Holder J.W., Zeng Q., Mahan A.E., Rha C.K., Sinskey A.J. (2010). Elucidation of β-Oxidation Pathways in Ralstonia Eutropha H16 by Examination of Global Gene Expression. J. Bacteriol..

[13] Riedel S.L., Lu J., Stahl U., Brigham C.J. (2014). Lipid and Fatty Acid Metabolism in Ralstonia Eutropha: Relevance for the Biotechnological Production of Value-Added Products. Appl. Microbiol. Biotechnol..

[14] Brandl H., Gross R.A., Lenz R.W., Fuller R.C. (1990). Plastics from Bacteria and for Bacteria: Poly (β-Hydroxyalkanoates) as Natural, Biocompatible, and Biodegradable Polyesters. Microb. Bioprod..

[15] Lafferty R.M. (1978). Microbial Production of Poly-(d-3-Hydroxybutyric Acid) 1981.

[16] Ramsay J.A., Berger E., Voyer R., Chavarie C., Ramsay B.A. (1994). Extraction of Poly-3-Hydroxybutyrate Using Chlorinated Solvents. Biotechnol. Tech..

[17] Ramsay J.A., Berger E., Ramsay B.A., Chavarie C. (1990). Recovery of Poly-3-Hydroxyalkanoic Acid Granules by a Surfactant-Hypochlorite Treatment. Biotechnol. Tech..

[18] Choi J., Lee S.Y. (1999). Efficient and Economical Recovery of Poly (3-hydroxybutyrate) from Recombinant Escherichia Coli by Simple Digestion with Chemicals. Biotechnol. Bioeng..

[19] Berger E., Ramsay B.A., Ramsay J.A., Chavarie C., Braunegg G. (1989). PHB Recovery by Hypochlorite Digestion of Non-PHB Biomass. Biotechnol. Tech..

[20] Hahn S.K., Chang Y.K., Kim B.S., Chang H.N. (1994). Optimization of Microbial Poly (3-hydroxybutyrate) Recover Using Dispersions of Sodium Hypochlorite Solution and Chloroform. Biotechnol. Bioeng..

[21] Zou Y., Yang M., Tao Q., Zhu K., Liu X., Wan C., Harder M.K., Yan Q., Liang B., Ntaikou I. (2023). Recovery of Polyhydroxyalkanoates (PHAs) Polymers from a Mixed Microbial Culture through Combined Ultrasonic Disruption and Alkaline Digestion. J. Environ. Manag..

[22] Zhila N., Kalacheva G., Volova T. (2015). Fatty Acid Composition and Polyhydroxyalkanoates Production by Cupriavidus Eutrophus B-10646 Cells Grown on Different Carbon Sources. Process Biochem..

[23] Koller M., Niebelschütz H., Braunegg G. (2013). Strategies for Recovery and Purification of Poly [(R)-3-hydroxyalkanoates](PHA) Biopolyesters from Surrounding Biomass. Eng. Life Sci..

[24] Manangan T., Shawaphun S. (2010). Quantitative Extraction and Determination of Polyhydroxyalkanoate Accumulated in Alcaligenes Latus Dry Cells. ScienceAsia.

[25] Braunegg G., Lefebvre G., Genser K.F. (1998). Polyhydroxyalkanoates, Biopolyesters from Renewable Resources: Physiological and Engineering Aspects. J. Biotechnol..

[26] Holmes P.A., Wright L.F., Alderson B., Senior P.J. (1982). A Process for the Extraction of Poly-3-Hydroxy-Butyric Acid from Microbial Cells.

[27] Reverchon E. (1997). Supercritical Fluid Extraction and Fractionation of Essential Oils and Related Products. J. Supercrit. Fluids.

[28] Hampson J.W., Ashby R.D. (1999). Extraction of Lipid-Grown Bacterial Cells by Supercritical Fluid and Organic Solvent to Obtain Pure Medium Chain-Length Polyhydroxyalkanoates. JAOCS J. Am. Oil Chem. Soc..

[29] Kobayashi D., Fujita K., Nakamura N., Ohno H. (2015). A Simple Recovery Process for Biodegradable Plastics Accumulated in Cyanobacteria Treated with Ionic Liquids. Appl. Microbiol. Biotechnol..

[30] Hejazi P., Vasheghani-Farahani E., Yamini Y. (2003). Supercritical Fluid Disruption of Ralstonia Eutropha for Poly(β-Hydroxybutyrate) Recovery. Biotechnol. Prog..

[31] Prat D., Hayler J., Wells A. (2014). A Survey of Solvent Selection Guides. Green Chem..

[32] Prat D., Wells A., Hayler J., Sneddon H., McElroy C.R., Abou-Shehada S., Dunn P.J. (2015). CHEM21 Selection Guide of Classical- and Less Classical-Solvents. Green Chem..

[33] Pagliano G., Galletti P., Samorì C., Zaghini A., Torri C. (2021). Recovery of Polyhydroxyalkanoates From Single and Mixed Microbial Cultures: A Review. Front. Bioeng. Biotechnol..

[34] de Souza Reis G.A., Michels M.H.A., Fajardo G.L., Lamot I., de Best J.H. (2020). Optimization of Green Extraction and Purification of PHA Produced by Mixed Microbial Cultures from Sludge. Water.

[35] Volova T., Shishatskaya E., Sevastianov V., Efremov S., Mogilnaya O. (2003). Results of Biomedical Investigations of PHB and PHB/PHV Fibers. Biochem. Eng. J..

[36] De Koning G.J.M., Kellerhals M., Van Meurs C., Witholt B. (1997). A Process for the Recovery of Poly (Hydroxyalkanoates) from Pseudomonads Part 2: Process Development and Economic Evaluation. Bioprocess Eng..

[37] Budde C.F., Riedel S.L., Willis L.B., Rha C., Sinskey A.J. (2011). Production of Poly (3-Hydroxybutyrate-Co-3-Hydroxyhexanoate) from Plant Oil by Engineered Ralstonia Eutropha Strains. Appl. Environ. Microbiol..

[38] Friedrich B., Hogrefe C., Schlegel H.G. (1981). Naturally Occurring Genetic Transfer of Hydrogen-Oxidizing Ability between Strains of Alcaligenes Eutrophus. J. Bacteriol..

[39] Santolin L., Waldburger S., Neubauer P., Riedel S.L. (2021). Substrate-Flexible Two-Stage Fed-Batch Cultivations for the Production of the PHA Copolymer P (HB-Co-HHx) with Cupriavidus Necator Re2058/PCB113. Front. Bioeng. Biotechnol..

[40] Oh S.J., Choi T.-R., Kim H.J., Shin N., Hwang J.H., Kim H.J., Bhatia S.K., Kim W., Yeon Y.J., Yang Y.-H. (2024). Maximization of 3-Hydroxyhexanoate Fraction in Poly (3-Hydroxybutyrate-Co-3-Hydroxyhexanoate) Using Lauric Acid with Engineered Cupriavidus Necator H16. Int. J. Biol. Macromol..

[41] Park Y.L., Bhatia S.K., Gurav R., Choi T.R., Kim H.J., Song H.S., Park J.Y., Han Y.H., Lee S.M., Park S.L. (2020). Fructose Based Hyper Production of Poly-3-Hydroxybutyrate from Halomonas Sp. YLGW01 and Impact of Carbon Sources on Bacteria Morphologies. Int. J. Biol. Macromol..

[42] Shin N., Kim S.H., Cho J.Y., Hwang J.H., Kim H.J., Oh S.J., Park S.-H., Park K., Bhatia S.K., Yang Y.-H. (2024). Fast Degradation of Polycaprolactone/Poly (Butylene Adipate-Co-Terephthalate) Blends by Novel Bacillus Strain NR4 with Broad Degrading Activity. J. Polym. Environ..

[43] Choi Y.K., Choi T.R., Gurav R., Bhatia S.K., Park Y.L., Kim H.J., Kan E., Yang Y.H. (2020). Adsorption Behavior of Tetracycline onto Spirulina Sp. (Microalgae)-Derived Biochars Produced at Different Temperatures. Sci. Total Environ..

[44] Choi T.R., Song H.S., Han Y.H., Park Y.L., Park J.Y., Yang S.Y., Bhatia S.K., Gurav R., Kim H.J., Lee Y.K. (2020). Enhanced Tolerance to Inhibitors of Escherichia Coli by Heterologous Expression of Cyclopropane-Fatty Acid-Acyl-Phospholipid Synthase (Cfa) from Halomonas Socia. Bioprocess Biosyst. Eng..

[45] Bernaerts T.M.M., Gheysen L., Kyomugasho C., Jamsazzadeh Kermani Z., Vandionant S., Foubert I., Hendrickx M.E., Van Loey A.M. (2018). Comparison of Microalgal Biomasses as Functional Food Ingredients: Focus on the Composition of Cell Wall Related Polysaccharides. Algal Res..

[46] Fei T., Cazeneuve S., Wen Z., Wu L., Wang T. (2016). Effective Recovery of Poly-β-Hydroxybutyrate (PHB) Biopolymer from Cupriavidus Necator Using a Novel and Environmentally Friendly Solvent System. Biotechnol. Prog..

[47] Murugan P., Gan C.-Y., Sudesh K. (2017). Biosynthesis of P (3HB-Co-3HHx) with Improved Molecular Weights from a Mixture of Palm Olein and Fructose by Cupriavidus Necator Re2058/PCB113. Int. J. Biol. Macromol..

[48] Van Nguyen T., Nagata T., Noso K., Kaji K., Masunaga H., Hoshino T., Hikima T., Sakurai S., Yamamoto K., Miura Y. (2021). Effect of the 3-Hydroxyhexanoate Content on Melt-Isothermal Crystallization Behavior of Microbial Poly (3-Hydroxybutyrate-Co-3-Hydroxyhexanoate). Macromolecules.

[49] Arcos-Hernández M.V., Laycock B., Donose B.C., Pratt S., Halley P., Al-Luaibi S., Werker A., Lant P.A. (2013). Physicochemical and Mechanical Properties of Mixed Culture Polyhydroxyalkanoate (PHBV). Eur. Polym. J..

[50] Selli F., Hufenus R., Gooneie A., Erdoğan U.H., Perret E. (2022). Structure–Property Relationship in Melt-Spun Poly (Hydroxybutyrate-Co-3-Hexanoate) Monofilaments. Polymers.

[51] Zurita-Paredes D., Flores-Bolaños D., Vizuete K., Debut A., Romero-Carvajal A. (2025). Solvent Dehydration and Low Temperature Vacuum Drying for SEM Imaging of Pre-Hatching Frog Embryos. J. Morphol..

[52] Gusnard D., Kirschner R.H. (1977). Cell and Organelle Shrinkage during Preparation for Scanning Electron Microscopy: Effects of Fixation, Dehydration and Critical Point Drying. J. Microsc..

[53] Rajaratanam D.D., Ariffin H., Hassan M.A., Kawasaki Y., Nishida H. (2017). Effects of (R)-3-Hydroxyhexanoate Units on Thermal Hydrolysis of Poly ((R)-3-Hydroxybutyrate-Co-(R)-3-Hydroxyhexanoate) s. Polym. Degrad. Stab..

[54] Okolie O., Kumar A., Edwards C., Lawton L.A., Oke A., McDonald S., Thakur V.K., Njuguna J. (2023). Bio-Based Sustainable Polymers and Materials: From Processing to Biodegradation. J. Compos. Sci..

[55] Porfyris A., Vasilakos S., Zotiadis C., Papaspyrides C., Moser K., Van der Schueren L., Buyle G., Pavlidou S., Vouyiouka S. (2018). Accelerated Ageing and Hydrolytic Stabilization of Poly(Lactic Acid) (PLA) under Humidity and Temperature Conditioning. Polym. Test..

[56] Watanabe T., He Y., Fukuchi T., Inoue Y. (2001). Comonomer Compositional Distribution and Thermal Characteristics of Bacterially Synthesized Poly (3-hydroxybutyrate-co-3-hydroxyhexanoate) s. Macromol. Biosci..

[57] Akdoğan M., Çelik E. (2018). Purification and Characterization of Polyhydroxyalkanoate (PHA) from a Bacillus Megaterium Strain Using Various Dehydration Techniques. J. Chem. Technol. Biotechnol..

[58] Kučera F., Petruš J., Jančář J. (2019). The Structure-Hydrolysis Relationship of Poly(3-Hydroxybutyrate). Polym. Test..

[59] Del Oso M.S., Mauricio-Iglesias M., Hospido A. (2021). Evaluation and Optimization of the Environmental Performance of PHA Downstream Processing. Chem. Eng. J..

[60] Hu S., McDonald A.G., Coats E.R. (2013). Characterization of Polyhydroxybutyrate Biosynthesized from Crude Glycerol Waste Using Mixed Microbial Consortia. J. Appl. Polym. Sci..

[61] Stratta L., Capozzi L.C., Franzino S., Pisano R. (2020). Economic Analysis of a Freeze-Drying Cycle. Processes.

[62] Nowak D., Jakubczyk E. (2020). The Freeze-Drying of Foods—The Characteristic of the Process Course and the Effect of Its Parameters on the Physical Properties of Food Materials. Foods.

[63] Mina Z.P., Kaseke T., Fadiji T., Silue Y., Fawole O.A. (2025). Combined Oven/Freeze Drying as a Cost and Energy-Efficient Drying Method for Preserving Quality Attributes and Volatile Compounds of Carrot Slices. Front. Hortic..

[64] Kešelj K., Pavkov I., Radojčin M., Stamenković Z. (2017). Comparison of energy consumption in the convective and freeze drying of raspberries poređenje utroška energije za sušenje maline konvktivnim postupkom I sušenjem zamrzavanjem. J. Process. Energy Agric..

[65] Iranshahi K., Rubinetti D., Onwude D.I., Psarianos M., Schlüter O.K., Defraeye T. (2023). Electrohydrodynamic Drying versus Conventional Drying Methods: A Comparison of Key Performance Indicators. Energy Convers. Manag..

[66] Carré P., Citeau M., Dauguet S. (2018). Hot Ethanol Extraction: Economic Feasibility of a New and Green Process. OCL.

[67] Khalati E., Bangalore Ashok R.P., Oinas P. (2023). Energy-efficient and Cost-effective Separation Model for Solvent Recovery from Colloidal Lignin Particles Dispersion. Can. J. Chem. Eng..

[68] Potrich E., Miyoshi S.C., Machado P.F.S., Furlan F.F., Ribeiro M.P.A., Tardioli P.W., Giordano R.L.C., Cruz A.J.G., Giordano R.C. (2020). Replacing Hexane by Ethanol for Soybean Oil Extraction: Modeling, Simulation, and Techno-Economic-Environmental Analysis. J. Clean. Prod..

